# Identification of a novel signature based on unfolded protein response-related gene for predicting prognosis in bladder cancer

**DOI:** 10.1186/s40246-021-00372-x

**Published:** 2021-12-20

**Authors:** Ke Zhu, Liu Xiaoqiang, Wen Deng, Gongxian Wang, Bin Fu

**Affiliations:** 1 Department of Urology, The First Affiliated Hospital of Nanchang University, 17 Yongwaizheng Street, Nanchang, 330006 Jiangxi People’s Republic of China; 2 Jiangxi Institute of Urology, Nanchang, 330006 Jiangxi People’s Republic of China

**Keywords:** Unfolded protein response, Bladder cancer, Prognostic, Biomarker

## Abstract

**Background:**

The unfolded protein response (UPR) served as a vital role in the progression of tumors, but the molecule mechanisms of UPR in bladder cancer (BLCA) have been not fully investigated.

**Methods:**

We identified differentially expressed unfolded protein response-related genes (UPRRGs) between BLCA samples and normal bladder samples in the Cancer Genome Atlas (TCGA) database. Univariate Cox analysis and the least absolute shrinkage and selection operator penalized Cox regression analysis were used to construct a prognostic signature in the TCGA set. We implemented the validation of the prognostic signature in GSE13507 from the Gene Expression Omnibus database. The ESTIMATE, CIBERSORT, and ssGSEA algorithms were used to explore the correlation between the prognostic signature and immune cells infiltration as well as key immune checkpoints (PD-1, PD-L1, CTLA-4, and HAVCR2). GDSC database analyses were conducted to investigate the chemotherapy sensitivity among different groups. GSEA analysis was used to explore the potential mechanisms of UPR-based signature.

**Results:**

A prognostic signature comprising of seven genes (CALR, CRYAB, DNAJB4, KDELR3, CREB3L3, HSPB6, and FBXO6) was constructed to predict the outcome of BLCA. Based on the UPRRGs signature, the patients with BLCA could be classified into low-risk groups and high-risk groups. Patients with BLCA in the low-risk groups showed the more favorable outcomes than those in the high-risk groups, which was verified in GSE13507 set. This signature could serve as an autocephalous prognostic factor in BLCA. A nomogram based on risk score and clinical characteristics was established to predict the over survival of BLCA patients. Furthermore, the signature was closely related to immune checkpoints (PD-L1, CTLA-4, and HAVCR2) and immune cells infiltration including CD8^+^ T cells, follicular helper T cells, activated dendritic cells, and M2 macrophages. GSEA analysis indicated that immune and carcinogenic pathways were enriched in high-risk group.

**Conclusions:**

We identified a novel unfolded protein response-related gene signature which could predict the over survival, immune microenvironment, and chemotherapy response of patients with bladder cancer.

## Abstract (articlehighlights)

1. We first comprehensive investigated the expression, protein–protein interaction, and prognostic value of unfolded protein response-related gene in bladder cancer.
2. We constructed and validated a novel prognostic signature based on seven unfolded protein response-related genes, which was an independent prognostic factors in bladder cancer.
3. We revealed the correlation between the prognostic signature and tumor immunosuppressive microenvironment.
4. We investigated the relationship between the prognostic signature and sensitivity of common chemotherapy drugs.

## Introduction

Bladder cancer (BLCA) is one of the most common genitourinary cancers and leads to unfavorable outcomes and unsatisfactory quality of life worldwide. Smoking and occupational exposures are closely associated with the tumorigenesis and progression of BLCA [1]. At present, surgical treatment remains the main therapeutic strategy for patients with BLCA. Despite several achievements in surgical equipment and systemic therapy, incidence and mortality of BLCA remain unacceptable [2]. Therefore, investigating the molecular mechanisms and identifying novel biomarkers are of enormous clinical significance to BLCA research and management.

The unfolded protein response (UPR), a prevalent biological phenomenon in eukaryotic cells, is an intracellular signaling pathway of adaptive cell self-protection [3]. Endoplasmic reticulum (ER) homeostasis is constantly threatened by physiological demands and pathological insults such as ER calcium depletion, hypoxia, altered glycosylation, nutrient deprivation, oxidative stress, and DNA damage, which can disrupt the protein folding process and subsequently result in accumulation of unfolded or misfolded proteins in the ER [3–6]. To dispose the accumulation of the unfolded or misfolded proteins, a series of signal transduction pathways, also collectively known as UPR, were activated in the ER, to maintain ER homeostasis through altering transcriptional and translational programs [7–10]. Several studies have confirmed that UPR has been implicated in the tumorigenesis owing to the rapid proliferation of tumor cells [5, 11, 12]. The research progress of targeted UPR in cancer therapy has also attracted increasing attention [7, 13–16].

In the present study, we investigated the mRNA expression profiles of UPR-related genes from several public databases and established a novel UPR-related gene signature for predicting the outcomes and chemotherapy sensitivity of patients with BLCA. Furthermore, we also found that the UPR-based signature was closely correlated with immune cell infiltration and key immune checkpoints.

## Materials and methods

### Data sources

Unfolded protein response-related genes (UPRRGs) were obtained from UALCAN (ualcan.path.uab.edu/home) database. The mRNA sequencing and clinical data for patients with BLCA were downloaded from The Cancer Genome Atlas (TCGA, https://portal.gdc.cancer.gov/) database and included data for 19 normal bladder samples and 414 BLCA samples. In addition, GSE13507 [17], containing 165 primary BLCA samples, was used for verification studies.

### Identification of differentially expressed UPRRGs

We used limma package in R software to identify differentially expressed UPRRGs (DEUPRRGs) in the TCGA-BLCA dataset. UPRGs with |log2fold change (FC)|> 1 and false discovery rate (FDR) < 0.05 were considered significantly differentially expressed.

### Gene ontology and KEGG analyses

To further explore the potential molecular mechanisms in which the DEUPRRGs were involved, Gene Ontology (GO) and Kyoto Encyclopedia of Genes and Genomes (KEGG) pathway analyses of DELRGs were performed with packages (ggplot2, org.Hs.eg.db, enrichplot, and clusterProfiler) in R software. *P* < 0.05 was considered statistically significant.

### Protein–protein interaction (PPI) network

The STRING database (http://www.string-db.org/) was applied to acquire protein–protein interaction (PPI) information related to DEUPRRGs. Cytoscape software (version 3.7.2) was utilized to establish and visualize the PPI network.

### Construction and validation of the prognostic signature based on DEUPRRGs

To identify the DEUPRRGs associated with prognosis in patients with BLCA in the TCGA dataset, univariate Cox regression analysis was performed. Only DEUPRRGs with *p* < 0.05 were selected for subsequent construction of a risk signature with the least absolute shrinkage and selection operator (LASSO) Cox regression model through packages (glmnet and survival) in R software. The risk score was calculated with the following formula: risk scores=${\sum }_{i}^{n}{\mathrm{X}}_{i}\times {\mathrm{Y}}_{\mathrm{i}}$ (where X is the coefficient of the prognosis-related DEUPRRGs and Y is the expression of the relevant gene). BLCA patients were categorized into two groups (high-risk and low-risk) based on the median risk score. Kaplan–Meier analysis was used to compare the survival of BLCA patients between the two groups. Time-dependent receiver operating characteristic (ROC) curves were applied to evaluate the reliability of the constructed signature for predicting prognosis. In addition, GSE13507 was used to verify the performance of this prognostic signature via the same method.

### Construction of a nomogram

We investigated the correlation between clinicopathological characteristics and the prognostic signature. Univariate and multivariate Cox regression analyses were performed to ascertain whether this prognostic signature was an independent prognostic indicator. To predict the overall survival of BLAC patients at 3 and 5 years, we constructed a nomogram composed of risk scores and clinical variables with R package (rms). The consistency index was used to assess the accuracy of the nomogram. The calibration curve was utilized to visualize the performance of the nomogram.

### Gene set enrichment analysis

Gene set enrichment analysis (GSEA) was conducted to further explore the potential molecular mechanisms among different groups. FDR < 5% and *p* < 0.05 was considered statistically significant.

### Immune infiltration analyses

Given the importance of tumor immune microenvironment, ESTIMATE algorithm was conducted to evaluate the stromal score, ESTIMATE score, and immune score and investigate the relationship between risk score and tumor microenvironment. The Cell type Identification By Estimating Relative Subsets Of RNA Transcripts (CIBERSORT) algorithm was used to identify the immune cell fractions of 22 distinct leukocyte subsets between different groups. Furthermore, we also performed single-sample gene set enrichment analysis (ssGSEA) to evaluate the differences in immune-related pathways between the two groups via package (gsva) in R software. *P* values < 0.05 were considered statistically significant.

### Chemotherapy sensitivity prediction

To explore the difference of chemotherapy sensitivity between different groups, we used GDSC database to estimate the half maximal inhibitory concentration (IC50) of chemotherapy drugs for predicting the sensitivity of chemotherapy drugs by using the package (pRRophetic). *P* < 0.05 were considered statistically significant.

### Validation of seven prognostic genes

The mRNA expression and promoter methylation level of the seven UPR genes in the prognostic signature in normal tissues and cancer tissues were examined in the UALCAN database (http://ualcan.path.uab.edu/index.html).

### Immune infiltrates analysis of seven prognostic genes

The Tumor IMmune Estimation Resource (TIMER, https://cistrome.shinyapps.io/timer/) database was used to explore the relationship between the immune cell abundances and the seven prognostic genes in BLCA. To further validate the correlation of seven genes with immune cells markers, the Gene Expression Profiling Interactive Analysis (GEPIA, https://gepia.cancer-pku.cn) database was used. Statistical analysis was conducted by Spearman’s correlation.

### Statistical analyses

All these statistical analyses were conducted in R, version 3.6.2. Gene expression, clinical characteristics, immune cell infiltration, and risk score were compared between different groups through the Wilcoxon test. The difference in survival among different groups was evaluated by Kaplan–Meier curve. Independent prognostic analysis was performed by univariate and multivariate Cox regression. *P* < 0.05 was considered statistically significant.

## Results

### Differentially expressed UPRRGs and functions

**Fig. 1:**
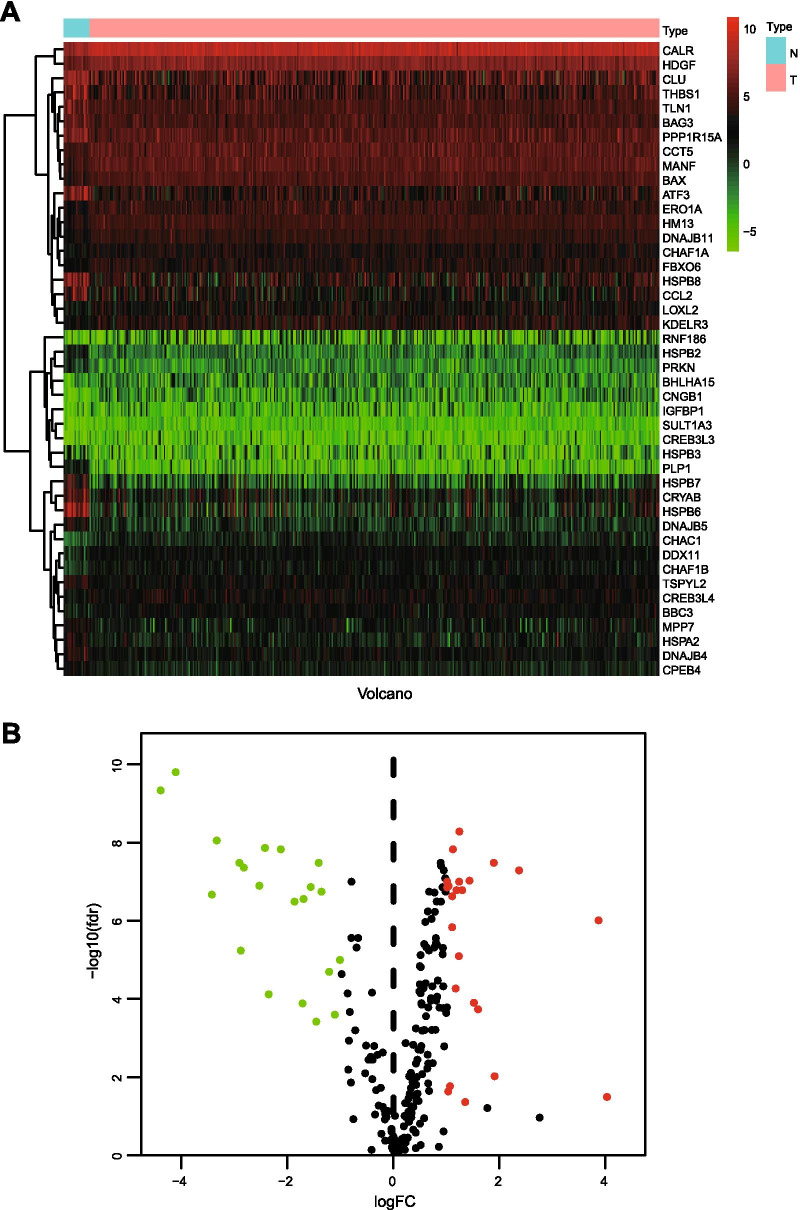
Differentially expressed UPRRGs in TCGA-BLCA set. **A** Heatmap showed differentially expressed UPRRGs; **B** volcano presented of differentially expressed UPRRGs, red showed the over-expression of UPR gene, and green showed the down-expression of UPR gene, FC: fold change, fdr: false discovery rate

**Fig. 2:**
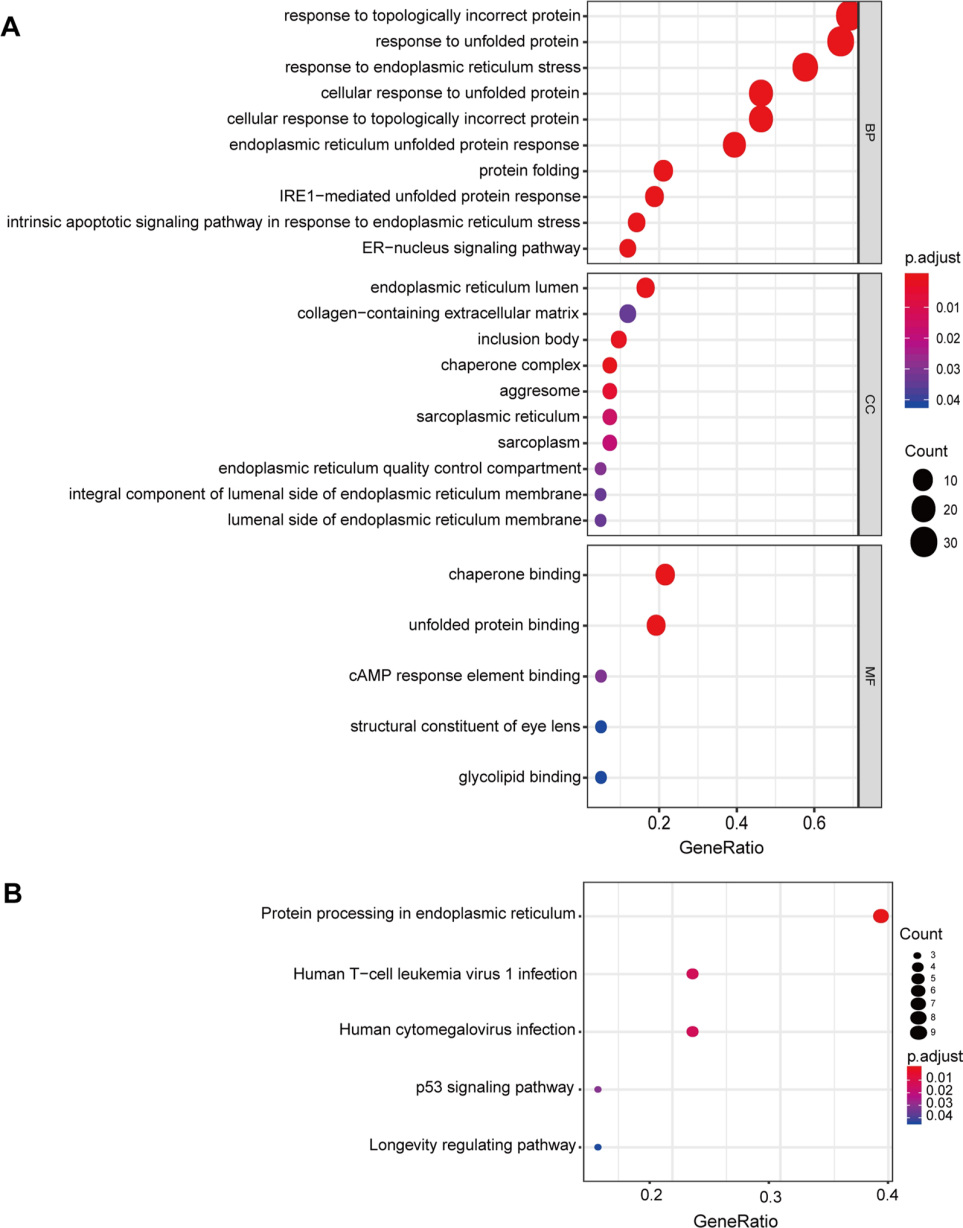
Enrichment analysis of differentially expressed UPRRGs. **A** GO analysis including biological process, cellular component, and molecular function; **B** KEGG analysis

We acquired 251 UPRRGs from the UALCAN database and analyzed the expression of these genes in BLCA samples compared with normal bladder tissues in the TCGA dataset. Forty-four UPRRGs were considered as differentially expressed genes in BLCA with the criteria of |log2 FC|> 1 and FDR < 0.05, including 21 downregulated genes and 23 upregulated genes (Fig. 1A, B). The results of GO analyses showed that the most enriched GO terms in the biological processes were response to topologically incorrect protein, response to unfolded protein, response to endoplasmic reticulum stress, cellular response to unfolded protein, and endoplasmic reticulum unfolded protein response (Fig. 2A). In the cellular component category, the DEUPRRGs were mainly enriched in endoplasmic reticulum lumen, collagen-containing extracellular matrix, inclusion body, and chaperone complex. In the molecular function category, chaperone binding, unfolded protein binding, cAMP response element binding, structural constituent of eye lens, and glycolipid binding were enriched in these DEUPRRGs. In the KEGG pathway analyses, the results showed that the DEUPRRGs were mainly involved in protein processing in the endoplasmic reticulum, human T-cell leukemia virus 1 infection, the p53 signaling pathway, and longevity regulating pathway (Fig. 2B).

### PPI network

**Fig. 3:**
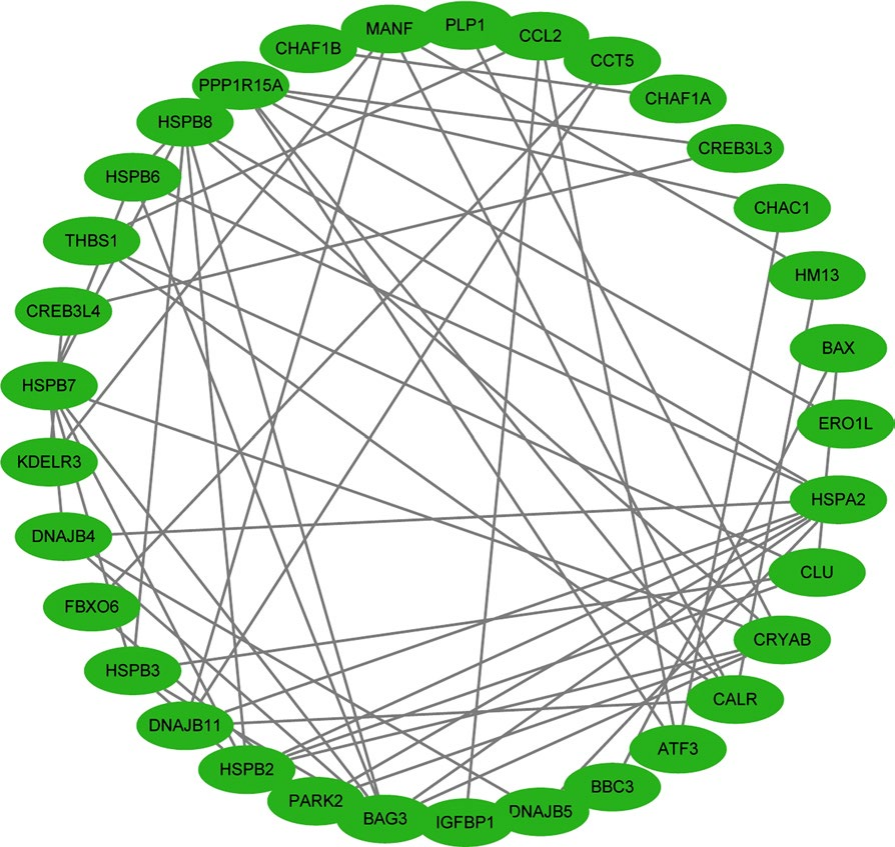
Protein–protein interaction network

To better understand the roles of these DEUPRRGs in regulating BLCA, a PPI network was constructed through the utilization of the STRING database. Then we used Cytoscape software to analyze and visualize the PPI network, which contained 33 nodes and 57 edges (Fig. 3).

### Construction and validation of a SLERGs-based signature

**Fig. 4:**
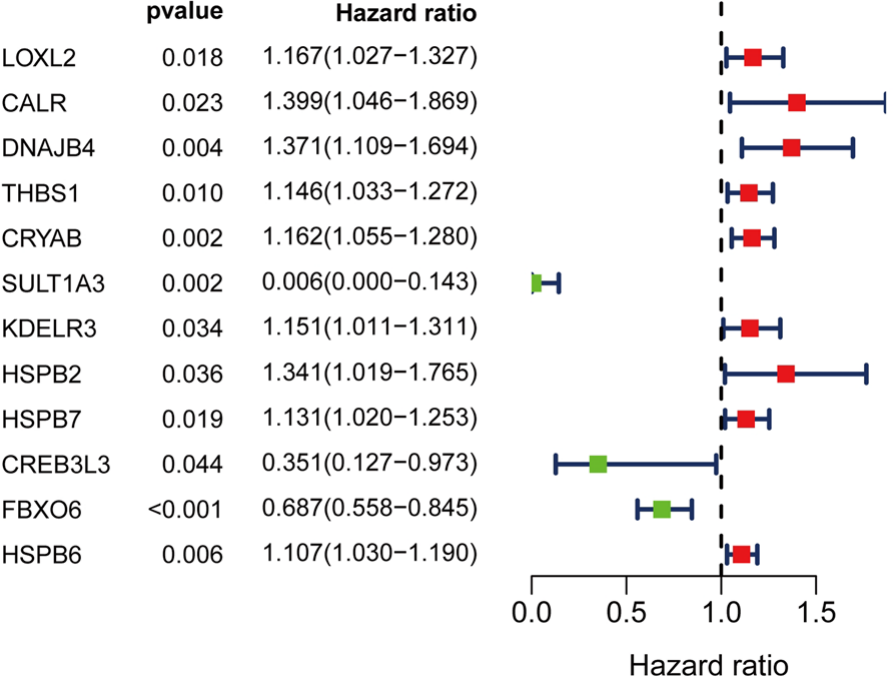
Univariate Cox regression analysis of differentially expressed UPRRGs

**Fig. 5:**
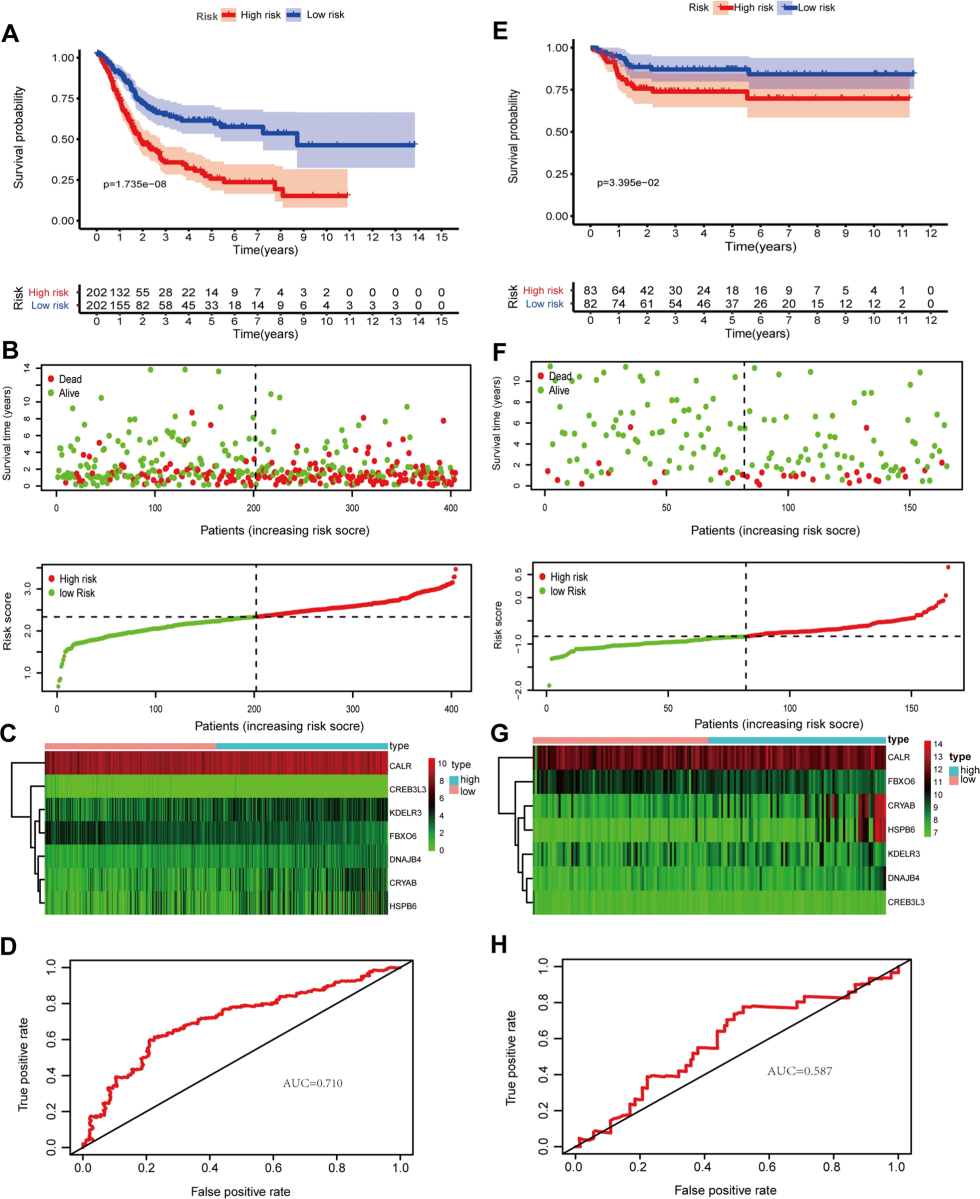
The correlation between the prognostic signature and outcome of BLCA patients. **A**–**E** Kaplan–Meier survival analysis of BLCA patients between high-risk groups and low-risk groups in TCGA set (**A**) and GSE13507 set (**E**); **B**–**F** distribution of survival status based on the median risk score in TCGA set (**B**) and GSE13507 set (**F**); **C**–**G** heatmap displayed the seven prognostic genes in TCGA set (**C**) and GSE13507 set (**G**); **D**–**H** time-independent receiver operating characteristic (ROC) analysis of risk scores predicting the overall survival in TCGA set (**D**) and GSE13507 set (**H**)

We used univariate Cox regression analysis to identify the DEUPRRGs closely correlated with the overall survival of BLCA patients. Eleven DEUPRRGs were selected for subsequent analyses (*p* < 0.05) (Fig. 4).We successfully constructed a prognostic model consisting of 7 DEUPRGs (CALR, CRYAB, DNAJB4, KDELR3, CREB3L3, HSPB6, and FBXO6) by performing LASSO Cox regression analysis based on the 11 candidate genes. The risk score was calculated as follows: Risk Score = CALR expression × 0.3265 + DNAJB4 expression × 0.2036 + CRYAB expression × 0.0609 + KDELR3 expression × 0.0715 − CREB3L3 expression × 0.5633 − FBXO6 expression × 0.3616 + HSPB6 expression × 0.0096. BLCA patients were classified into high- and low-risk groups based on the median risk score. The Kaplan–Meier curves showed that patients in the low-risk group had better outcomes than those in the high-risk group (Fig. 5A). The area under the ROC (AUC) curve value of the constructed model was 0.710 at 5 years (Fig. 5D). The survival status of each patient was represented on dot plots, which showed that patients in the low-risk group had more favorable outcomes than those in the high-risk group (Fig. 5B). Figure 5C indicates that the high-risk group highly expressed CRYAB, DNAJB4, HSPB6, KDELR3, and CALR, while the low-risk group highly expressed FBXO6 and CREB3L3. We also verified the prognostic capacity of this prognostic signature in the Gene Expression Omnibus (GEO) dataset, showing consistent results between GEO dataset and TCGA dataset (Fig. 5E, F, H). These results indicated acceptable performance of the prognostic signature.

### Construction of a nomogram

**Fig. 6:**
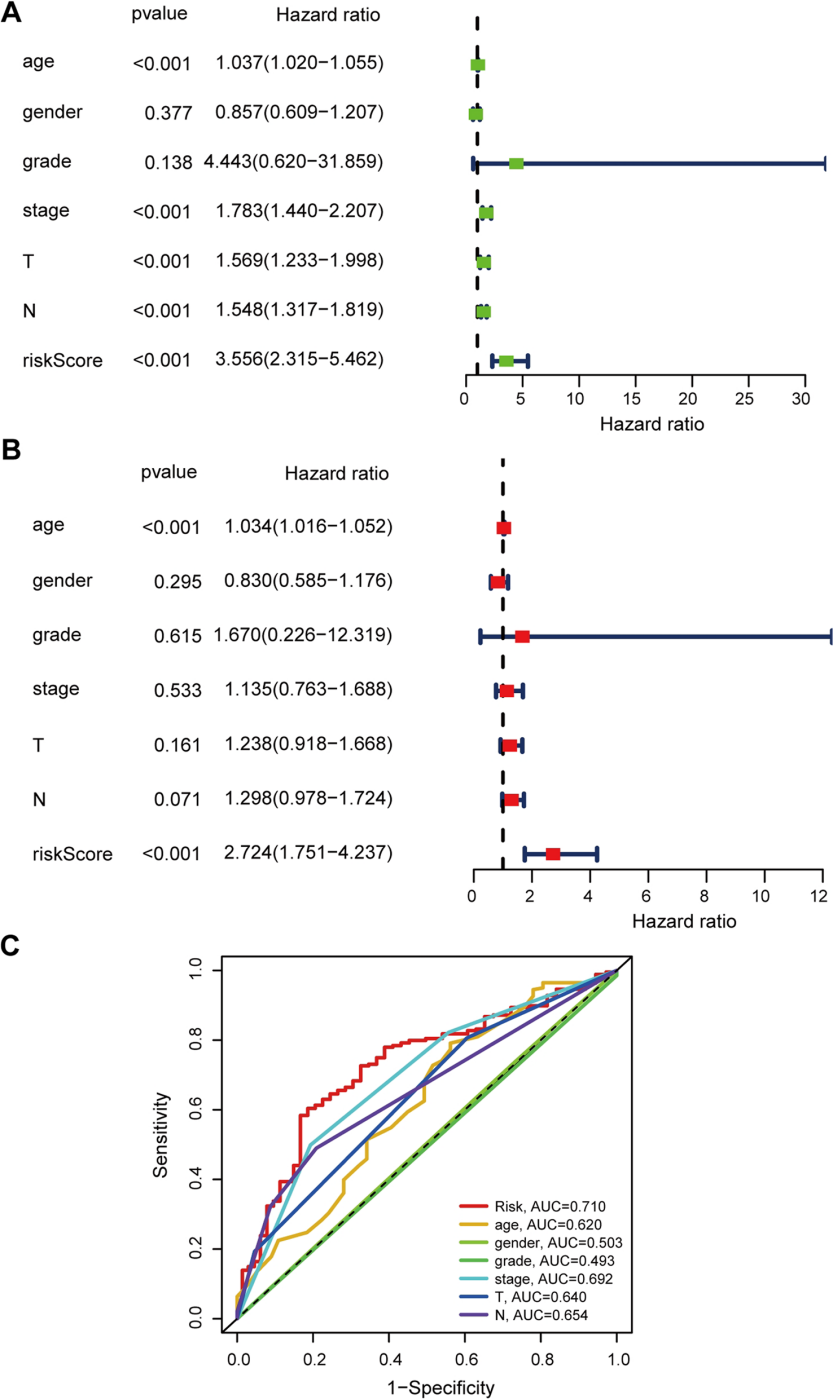
The prognostic signature was an independent prognostic factor for BLCA in TCGA set. **A** The correlations between the risk score for OS and clinicopathological factors by univariate Cox regression analysis; **B** the correlations between the risk score for OS and clinicopathological factors by multivariate Cox regression analysis; **C** ROC curves of the clinical characteristics and risk score

**Fig. 7:**
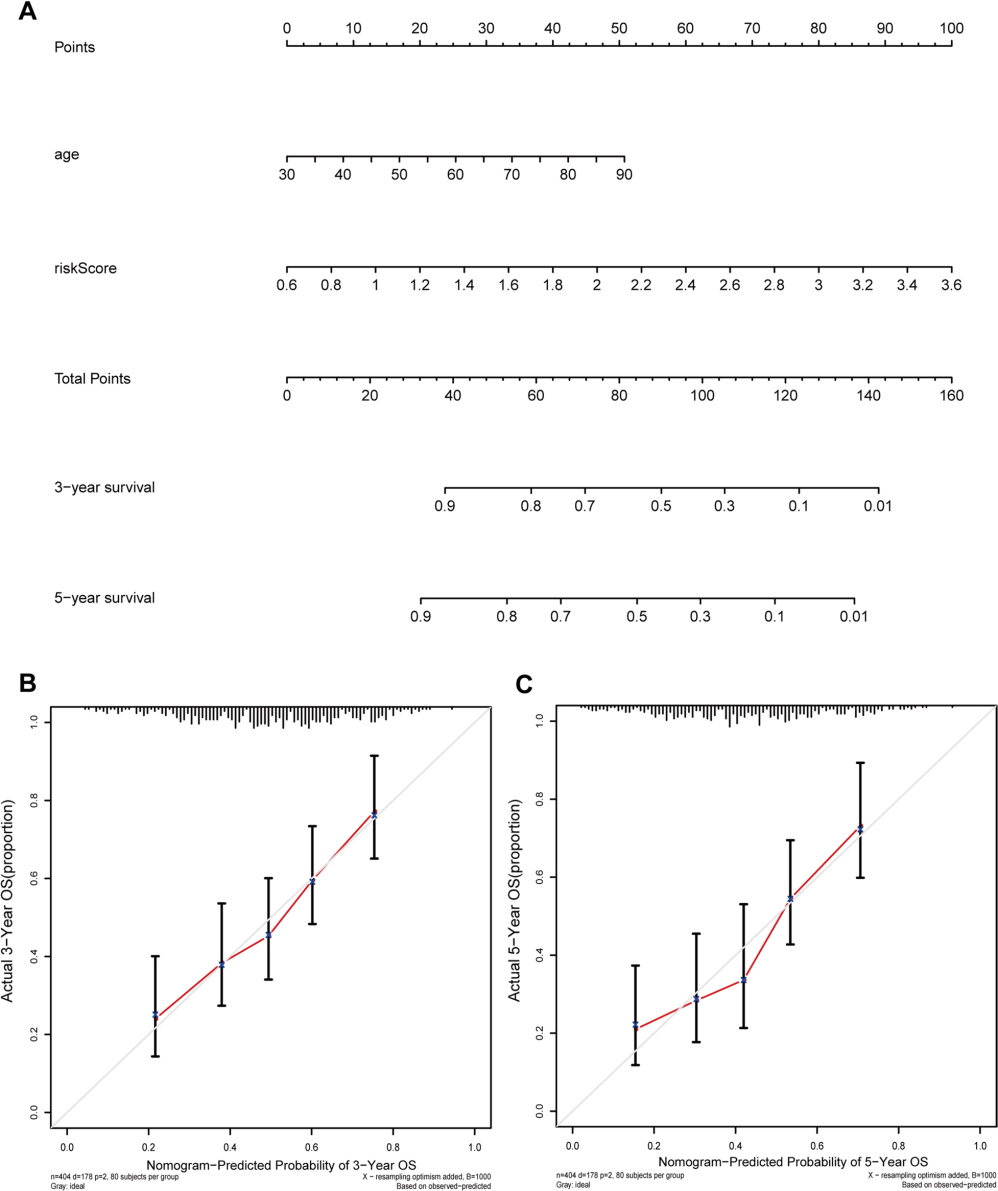
Construction of a nomogram. **A** Nomogram for predicting 3‐ or 5‐year OS; **B** the calibration plots for predicting 3‐year OS; **C** the calibration plots for predicting 5‐year OS

Univariate and multivariate Cox proportional hazard models were performed to identify the prognostic variables. Based on the univariate analysis, age (HR 1.037; 95% CI 1.020–1.055; *p* < 0.001), TNM stage (HR 1.783; 95% CI 1.440–2.207; *p* < 0 0.001), T stage (HR 1.569; 95% CI 1.233–1.998; *p* < 0 0.001), N stage (HR 1.548; 95% CI 1.317–1.819; *p* < 0.001), and risk score (HR 3.556; 95% CI 2.315–5.462; *p* < 0.001) were believed to be related to unfavorable outcomes (Fig. 6A). In the multivariate analysis, age (HR 1.034; 95% CI 1.016–1.052; *p* < 0.001) and risk score (HR 2.724; 95% CI 1.751–4.238; *p* < 0.001) could serve as independent prognostic factors (Fig. 6B). To further predict the outcomes of BLCA patients, we constructed a nomogram consisting of the risk score and age. The nomogram predicted the 3- and 5-year survival rates of patients with BLCA (Fig. 7A). The calibration curve showed that the actual patient outcomes were in keeping with the predicted outcomes (Fig. 7B, C). The C index of the nomogram was 0.645, which indicated the definite predictive ability of the nomogram.

### The correlation between the prognostic signature and clinical characteristics

**Fig. 8:**
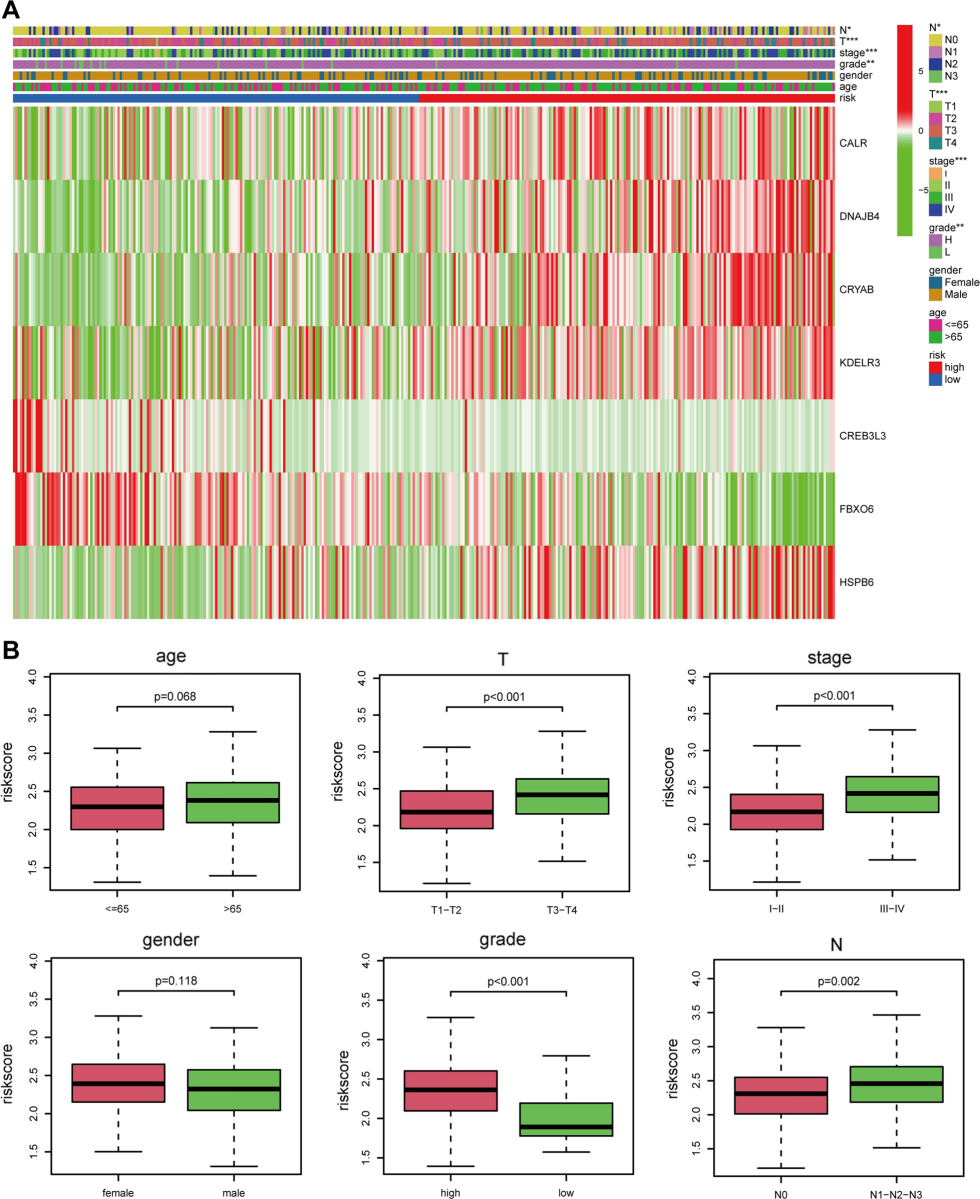
The correlation between the signature and clinical characteristics. **A** Heatmap showed the correlation between the risk scores and clinicopathological factors. The elevated risk score was closely correlated with several inferior clinical characteristics (TNM stage, T stage, N stage, and grade). *P*: 0.05 > * > 0.01 > ** > 0.001 > ***. **B** Boxplot showed the correlation between the risk scores and clinicopathological factors

**Fig. 9:**
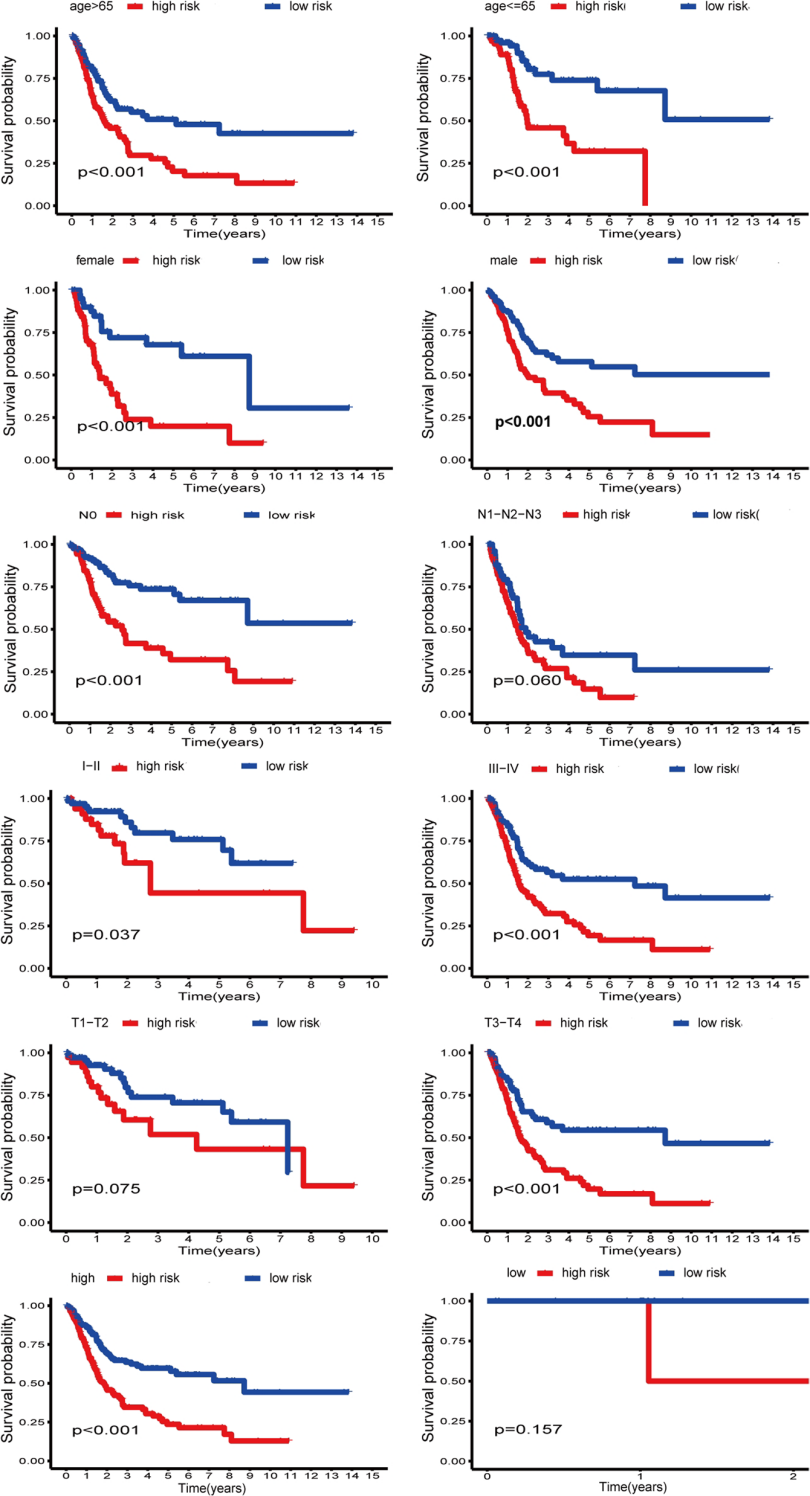
Subgroup analysis

We also explored the relationship between the prognostic signature and clinical characteristics. The results showed that the elevated risk score was closely correlated with several inferior clinical characteristics (TNM stage, T stage, N stage, and grade) (Fig. 8A, B). With an increase in risk score, the expression levels of CALR, CRYAB, DNAJB4, KDELR3, and HSPB6 were increased, while the expression levels of FBXO6 and CREB3L3 were decreased. In addition, the elevated expressions of CALR, CRYAB, DNAJB4, KDELR3, and HSPB6 were associated with inferior clinical characteristics, while the under-expressions of FBXO6 and CREB3L3 were associated with superior clinical characteristics. Furthermore, the signature was related to unfavorable prognosis in certain subgroups stratified by sex (male or female), age (> 65 years) or (≤ 65 years), N stage (N0), TNM stage (I-II) or (III-IV), grade (high), and T stage (T3-T4) (Fig. 9). However, the signature showed no differences in other subgroups of N stage (N1–N2–N3), grade (low), and T stage (T1–T2).

### GSEA

**Fig. 10:**
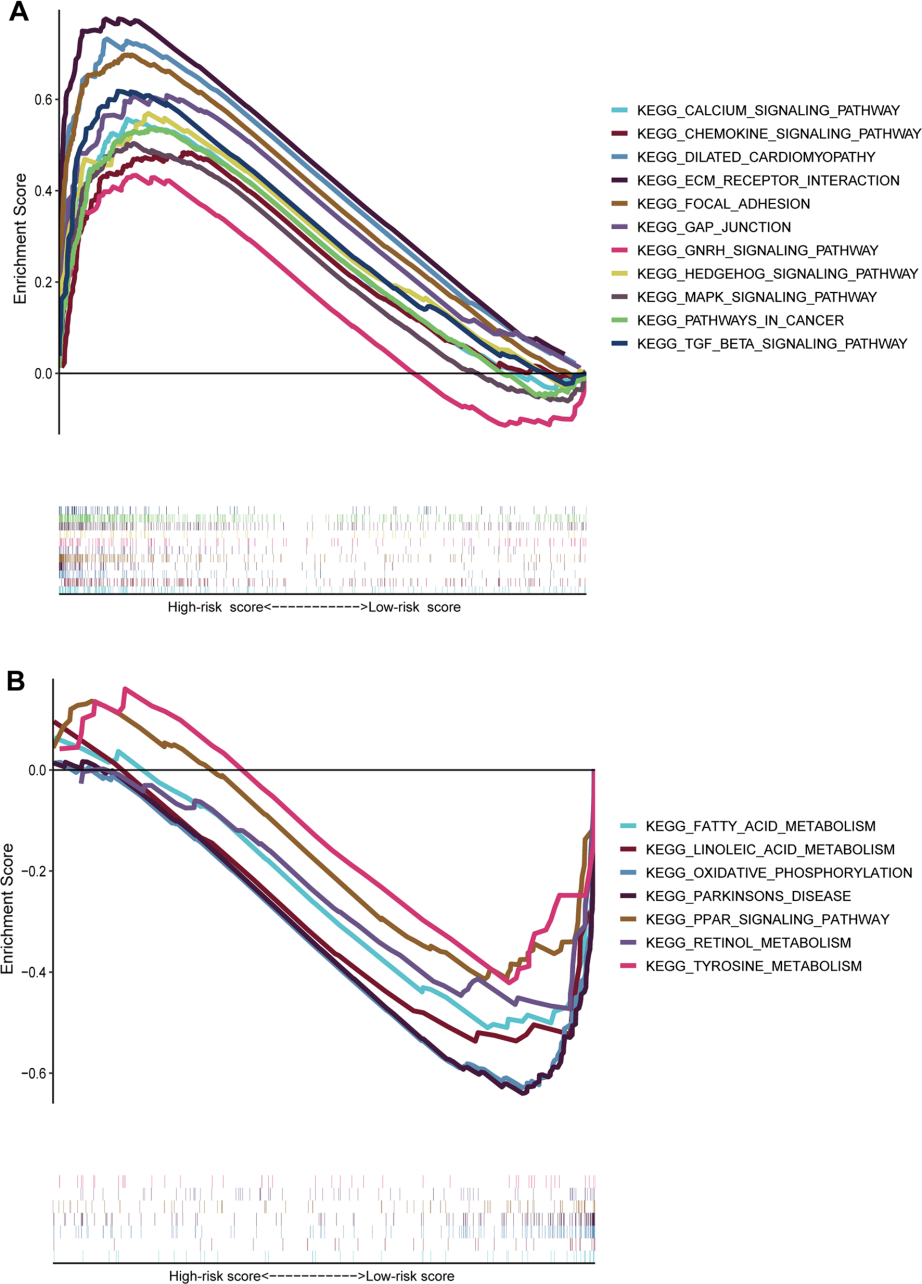
GSEA analysis between high-risk group (**A**) and low-risk group (**B**)

We performed GSEA to explore the signaling pathways that might be closely enriched among two groups. The results of GSEA indicated that focal adhesion, dilated cardiomyopathy, ECM receptor interaction, TGF-β signaling pathway, GAP junction, pathway in cancer, calcium signaling pathway, MAPK signaling pathway, chemokine signaling pathway, GnRH signaling pathway, and hedgehog signaling pathway were mainly enriched in high-risk group (Fig. 10A). In contrast, oxidative phosphorylation, retinol metabolism, fatty acid metabolism, linoleic acid metabolism, tyrosine metabolism, PPAR signaling pathway, and Parkinson's disease were mainly enriched in low-risk group (Fig. 10B).

### Immune cell infiltration analyses

**Fig. 11:**
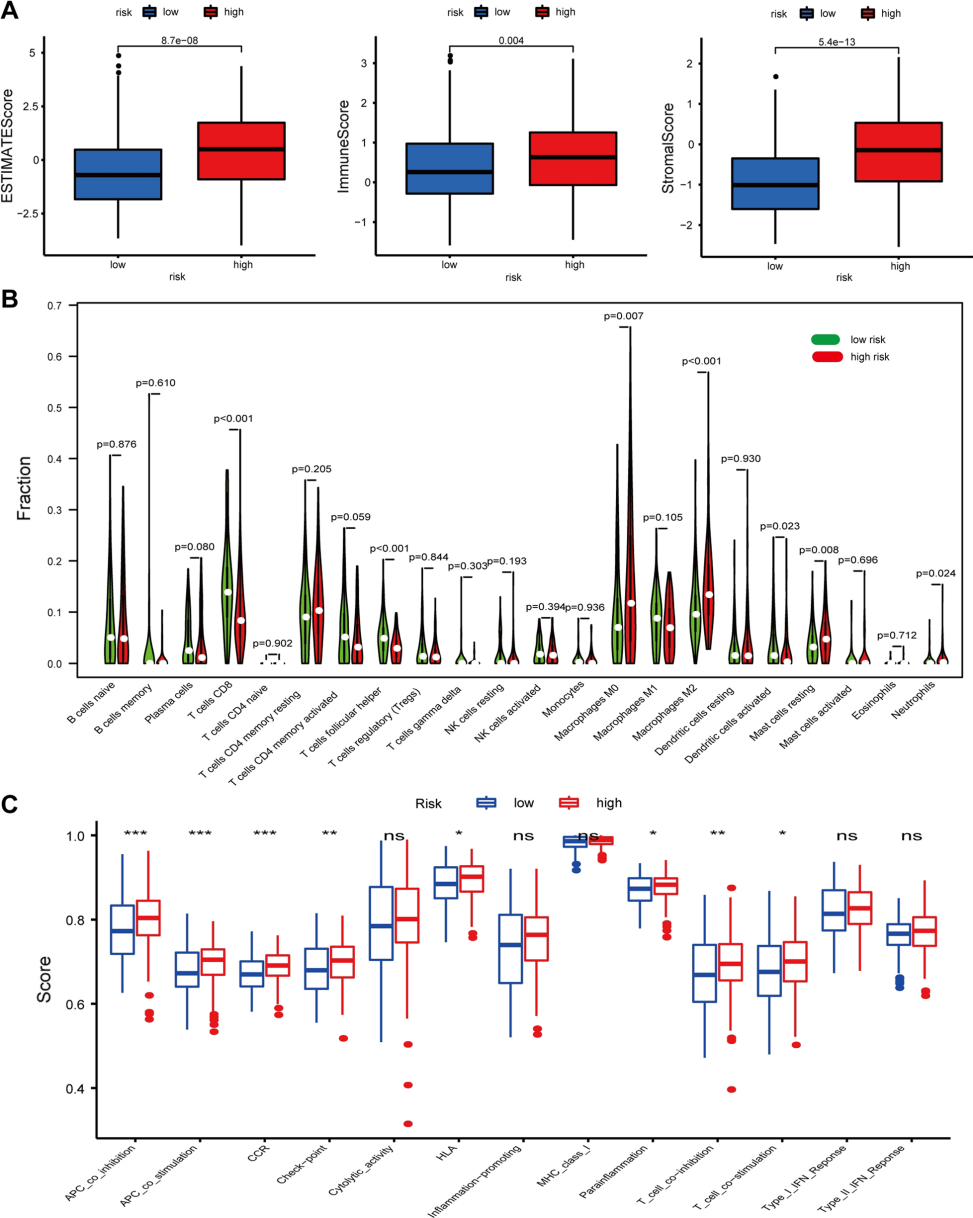
Immune cells infiltration and correlated pathways between high-risk groups and low-risk groups. **A** Immune microenvironment analysis between high-risk groups and low-risk groups by ESTIMATE; **B** immune cells infiltration between high-risk groups and low-risk groups by CIBERSORT; **C** immune-related pathways between high-risk groups and low-risk groups by ssGSEA. *P*: 0.05 > * > 0.01 > ** > 0.001 > ***

**Fig. 12:**
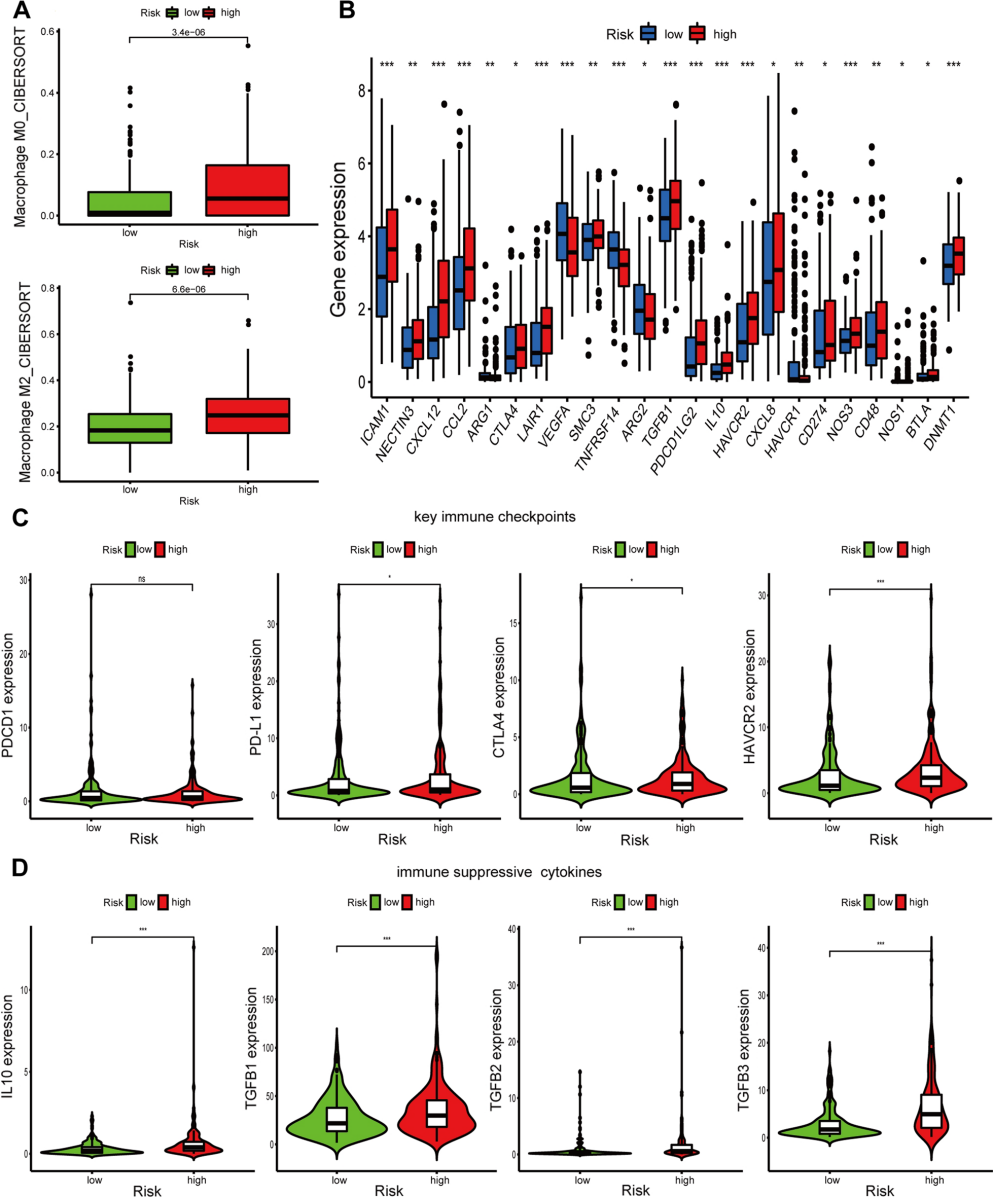
The correlation between immune microenvironment and the prognostic signature. **A** Immune suppressive cells between high-risk groups and low-risk groups; **B** immune suppressive molecules between high-risk groups and low-risk groups; **C** key immune checkpoints between high-risk groups and low-risk groups; **D** immunosuppressive chemokines between high-risk groups and low-risk groups. *P*: 0.05 > * > 0.01 > ** > 0.001 > ***

To further investigate the relationship between risk score and immune cell infiltration, ESTIMATE algorithm was first performed. We found that stromal score, immune score, and ESTIMATE score were remarkably higher in high-risk group than those in low-risk group (Fig. 11A). In addition, the results of the CIBERSORT algorithm showed the significant differences in the proportions of distinct leukocyte subsets between different groups. The proportions of CD8^+^ T cells, follicular helper T cells, and activated dendritic cells were higher in the low-risk group, whereas those of M0 macrophages, M2 macrophages, and neutrophils were higher in the high-risk group (Fig. 11B). Furthermore, the ssGSEA results indicated that a majority of immune-related pathways had higher enrichment scores in the high-risk group than in the low-risk group. Cytolytic activity, inflammation promotion, MHC class I, type I IFN response, and type II IFN response were not significantly different between the two groups (Fig. 11C). CIBERSORT results showed that immune suppressive cells were mainly enriched in high-risk group (Fig. 12A). To verify the immunosuppressive status of high-risk group, we further investigated the expression of immune suppressive molecules among different groups. The results indicated that immune suppressive molecules were elevated in high-risk group, suggesting that patients of high-risk group have inferior activities of anticancer immune response (Fig. 12B). Furthermore, we also analyzed the expression of key immune checkpoints (PDCD1, PD-L1(CD274), CTLA-4, and HAVCR2) and found that PD-L1, CTLA-4, and HAVCR2 were upregulated in high-risk group (Fig. 12C). In addition, chemokines implicated in immunosuppressive process (IL-10, TGF-β1, TGF-β2, TGF-β3) were also elevated upregulated in high-risk group (Fig. 12D). All these results indicated that the unfavorable outcomes of high-risk patients might be owing to the immunosuppressive microenvironment.

### TIMER analysis

**Fig. 13:**
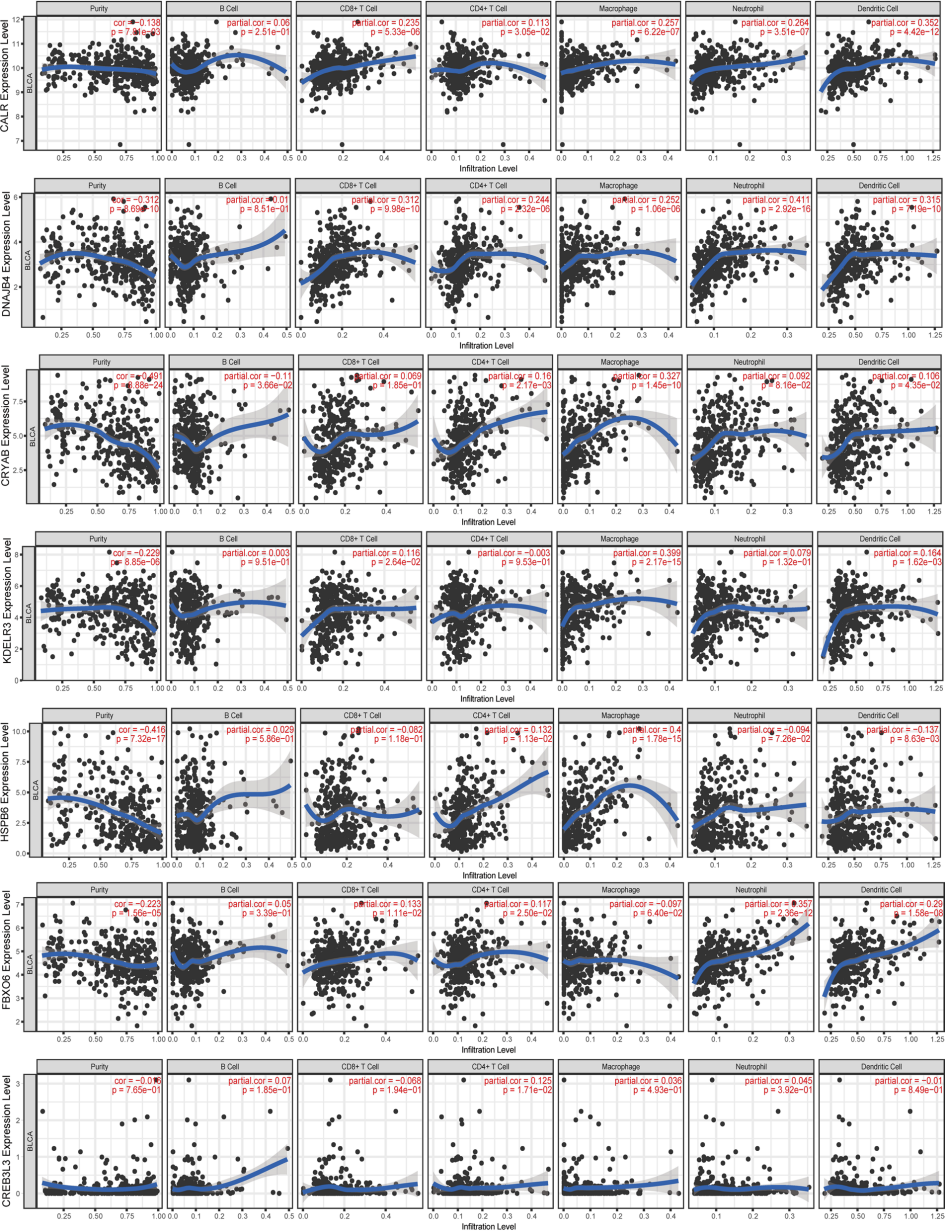
Immune infiltrates analysis of seven prognostic genes by TIMER database

**Fig. 14:**
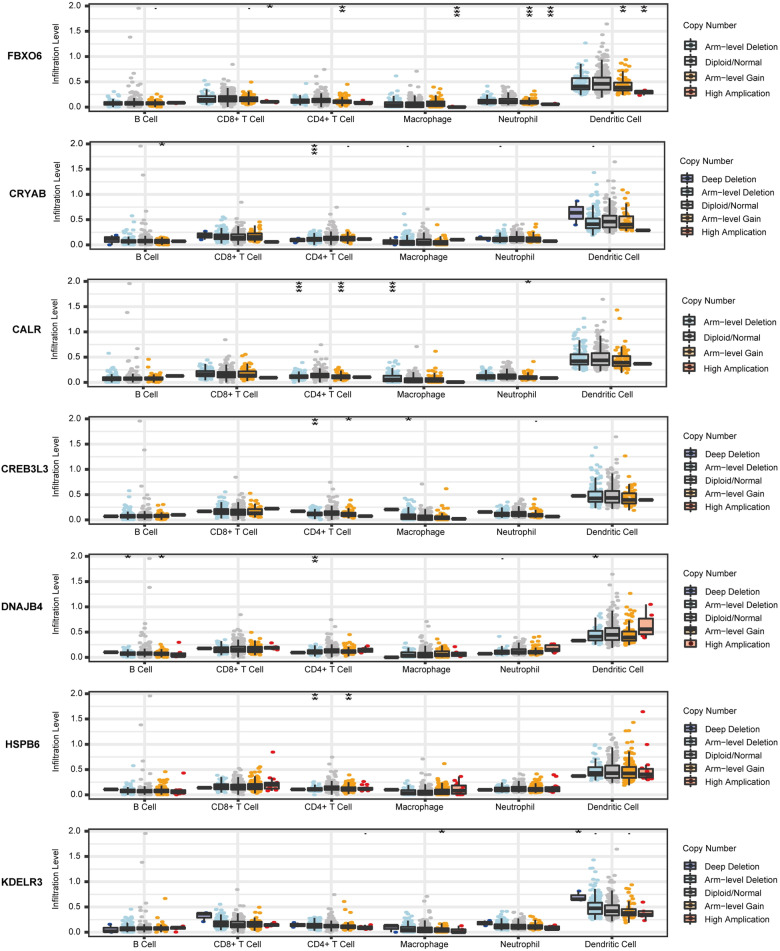
The relationship between immune cells infiltration and CNVs of seven prognostic genes by TIMER database. *P*: 0.05 > * > 0.01 > ** > 0.001 > ***

The TIMER database was used to investigate the correlation between the mRNA expressions of CALR, CREB3L3, HSPB6, FBXO6, CRYAB, DNAJB4, KDELR3, and the abundances of immune cells in BLCA (Fig. 13). The expression level of CALR showed significant correlations with the abundances of CD8^+^ T cells (cor = 0.235, *p* = 5.33e−6), CD4^+^ T cell (cor = 0.113, *p* = 3.05e−2), macrophages (cor = 0.257, *p* = 6.22e−7), neutrophils (cor = 0.264, *p* = 3.51e−7), and dendritic cells (cor = 0.352, *p* = 5.33e−12). DNAJB4 was closely related to the CD8^+^ T cells (cor = 0.312, *p* = 9.98e−10), CD4^+^ T cell (cor = 0.244, *p* = 0.244e−6), macrophages (cor = 0.252, *p* = 1.02e−7), neutrophils (cor = 0.411, *p* = 2.92e−16), and dendritic cells (cor = 0.315, *p* = 7.19e−10). CRYAB showed strong correlations with B cells (cor = − 0.11, *p* = 3.66e−2), CD4^+^ T cells (cor = 0.16, *p* = 2.17e−3), macrophages (cor = 0.327, *p* = 1.45e−10), and dendritic cells (cor = 0.106, *p* = 4.35e−2). The expression of KDELR3 displayed significant correlation with the abundance of CD8^+^ T cells (cor = 0.116, *p* = 2.64e−2), macrophages (cor = 0.399, *p* = 2.17e−15), and dendritic cells (cor = 0.164, *p* = 1.62e−3). CREB3L3 was only related to CD4^+^ T cells (cor = 0.125, *p* = 1.17e−2). FBXO6 showed significant correlation with the abundances of CD8^+^ T cells (cor = 0.133, *p* = 1.11e−2), neutrophils (cor = 0.357, *p* = 2.36e−12), and dendritic cells (cor = 0.29, *p* = 1.58e−8). HSPB6 showed strong correlations with the abundances of CD4^+^ T cells (cor = 0.132, *p* = 1.13e−2), macrophages (cor = 0.4, *p* = 1.78e−15), and dendritic cells (cor = -0.137, *p* = 8.63e−3). Meanwhile, we found that seven UPRGs CNVs were correlated with several immune cells (Fig. 14). We also revealed the correlation between seven UPRGs and immune marker genes of immune cells by using GEPIA database. The results suggested that seven UPRGs were also related to immune marker genes using the GEPIA database (Table 1).

**Table 1:** The correlation between seven UPR genes and immune cell markers

Description	Gene markers	CRYAB		CALR		DNAJB4		HSPB6		KDELR3		FBXO6		CREB3L3	
		GEPIA													
		*R*	*P*	*R*	*P*	*R*	*P*	*R*	*P*	*R*	*P*	*R*	*P*	*R*	*P*
B cell	CD19	0.41	***	0.12	*	0.13	**	0.46	***	0.27	***	0.24	***	0.15	**
	CD79A	0.42	***	0.056	ns	0.14	**	0.46	***	0.25	***	0.18	***	0.11	*
	MS4A1	0.36	***	− 0.018	ns	0.17	***	0.46	***	0.19	***	0.13	*	0.16	**
CD8^+^ T cell	CD8A	0.28	***	0.24	***	0.3	***	0.18	***	0.18	***	0.46	***	0.1	*
	CD8B	0.23	***	0.27	***	0.25	***	0.19	***	0.16	**	0.39	***	0.08	ns
	IL2RA	0.45	***	0.31	***	0.41	***	0.32	***	0.3	***	0.38	***	0.12	*
M1 macrophages	PTGS2	0.16	**	0.11	*	0.34	***	0.12	*	0.22	***	0.13	*	0.072	ns
	NOS2	0.12	*	− 0.081	ns	0.1	*	0.26	***	0.11	*	0.14	**	0.13	**
M2 macrophages	CD163	0.54	***	0.36	***	0.35	***	0.44	***	0.42	***	0.34	***	0.094	ns
	CD209	0.52	***	0.29	***	0.43	***	0.39	***	0.33	***	0.31	***	0.085	ns
	MRC1	0.51	***	0.28	***	0.39	***	0.4	***	0.39	***	0.29	***	0.11	*
M0 macrophages	CD14	0.56	***	0.35	***	0.38	***	0.39	***	0.31	***	0.33	***	0.057	ns
	CD33	0.53	***	0.31	***	0.34	***	0.45	***	0.33	***	0.29	***	0.11	*
	ITGAX	0.53	***	0.26	***	0.41	***	0.41	***	0.36	***	0.34	***	0.12	*
TAM	CCL2	0.54	***	0.23	***	0.34	***	0.52	***	0.41	***	0.27	***	0.14	**
	CD68	0.31	***	0.29	***	0.44	***	0.14	**	0.11	*	0.37	***	0.0091	ns
	CD86	0.5	***	0.31	***	0.45	***	0.32	***	0.26	***	0.38	***	0.083	ns
Neutrophil	CD55	0.13	**	0.014	ns	0.21	***	0.14	**	0.34	***	0.016	ns	0.07	ns
	FCGR3A	0.49	***	0.37	***	0.4	***	0.34	***	0.39	***	0.39	***	0.094	ns
	ITGAM	0.5	***	0.33	***	0.43	***	0.39	***	0.37	***	0.35	***	0.15	**
Natural killer cell	CD7	0.35	***	0.27	***	0.29	***	0.24	***	0.19	***	0.47	***	0.082	ns
	KIR3DL1	0.19	***	0.16	**	0.19	***	0.14	**	0.14	**	0.33	***	0.11	*
Dendritic cell	CD1C	0.37	***	0.0072	ns	0.26	***	0.36	***	0.0078	ns	0.018	ns	0.048	ns
	THBD	0.25	***	0.057	ns	0.32	***	0.048	ns	− 0.038	ns	0.16	**	− 0.17	***
T cell exhaustion	CTLA4	0.36	***	0.26	***	0.31	***	0.2	***	0.19	***	0.43	***	0.12	*
	LAG3	0.31	***	0.36	***	0.28	***	0.15	**	0.21	***	0.47	***	0.12	*
	PD-1	0.32	***	0.27	***	0.29	***	0.23	***	0.21	***	0.48	***	15	**
Treg	FOXP3	0.39	***	0.24	***	0.38	***	0.29	***	0.23	***	0.39	***	0.14	**
	IL7R	0.51	***	0.27	***	0.42	***	0.35	***	0.35	***	0.3	***	0.075	ns
	NT5E	0.4	***	0.25	***	0.39	***	0.18	***	0.27	***	0.16	**	− 0.024	ns
Th1 cell	CCR1	0.4	***	0.36	***	0.36	***	0.32	***	0.41	***	0.34	***	0.12	*
	CCR5	0.37	***	0.3	***	0.35	***	0.28	***	0.29	***	0.43	***	0.15	**
	IL12RB1	0.37	***	0.28	***	0.34	***	0.25	***	0.22	***	0.46	***	0.11	*
Th2 cell	CCR4	0.35	***	0.081	ns	0.38	***	0.39	***	0.25	***	0.26	***	0.17	***
	CCR8	0.34	***	0.14	**	0.46	***	0.25	***	0.28	***	0.31	***	0.18	***

***P < 0.001, **P < 0.01, *P < 0.05, ns

### Validation of the mRNA expression of 7 prognostic genes

**Fig. 15:**
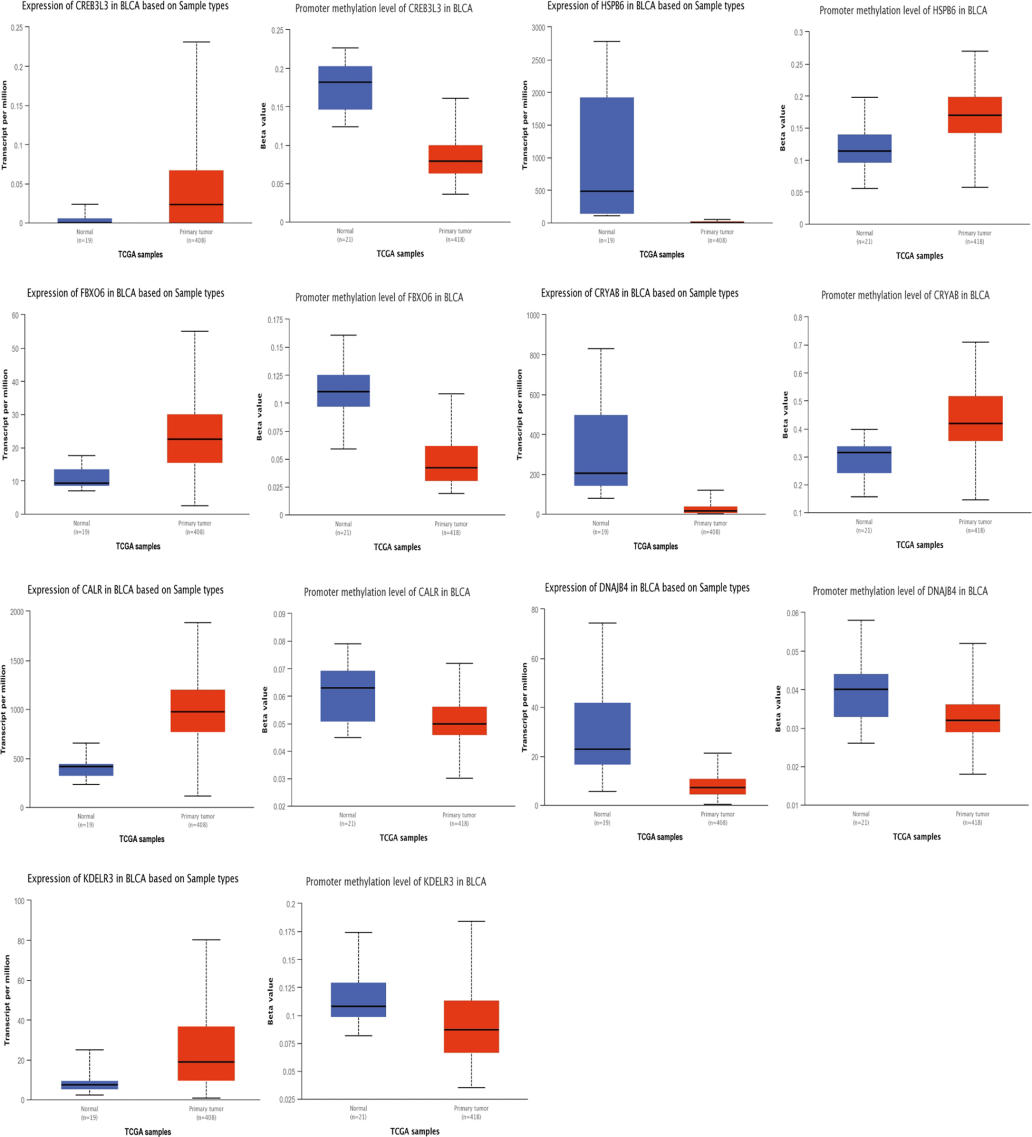
Validation of the mRNA expression and promoter methylation of seven prognostic genes

UALCAN database was used to examine the mRNA expression and promoter methylation level of seven UPR genes (Fig. 15). The results of UALCAN database analysis showed that the mRNA expression levels of CALR, CREB3L3, FBOX6, and KDELR3 were increased in cancer tissues compared with normal tissues, while the mRNA expression levels of CRYAB, DNAJB4, and HSPB6 were decreased in BLCA. Furthermore, the elevated expressions of CALR, CREB3L3, FBOX6, and KDELR3 and the down-expressions of CRYAB, and HSPB6 might be correlated with promoter methylation. However, we did not find the correlation between the mRNA expression and promoter methylation level of DNAJB4. All these results were consistent with previous results.

### Correlation between risk score and chemotherapy sensitivity

**Fig. 16:**
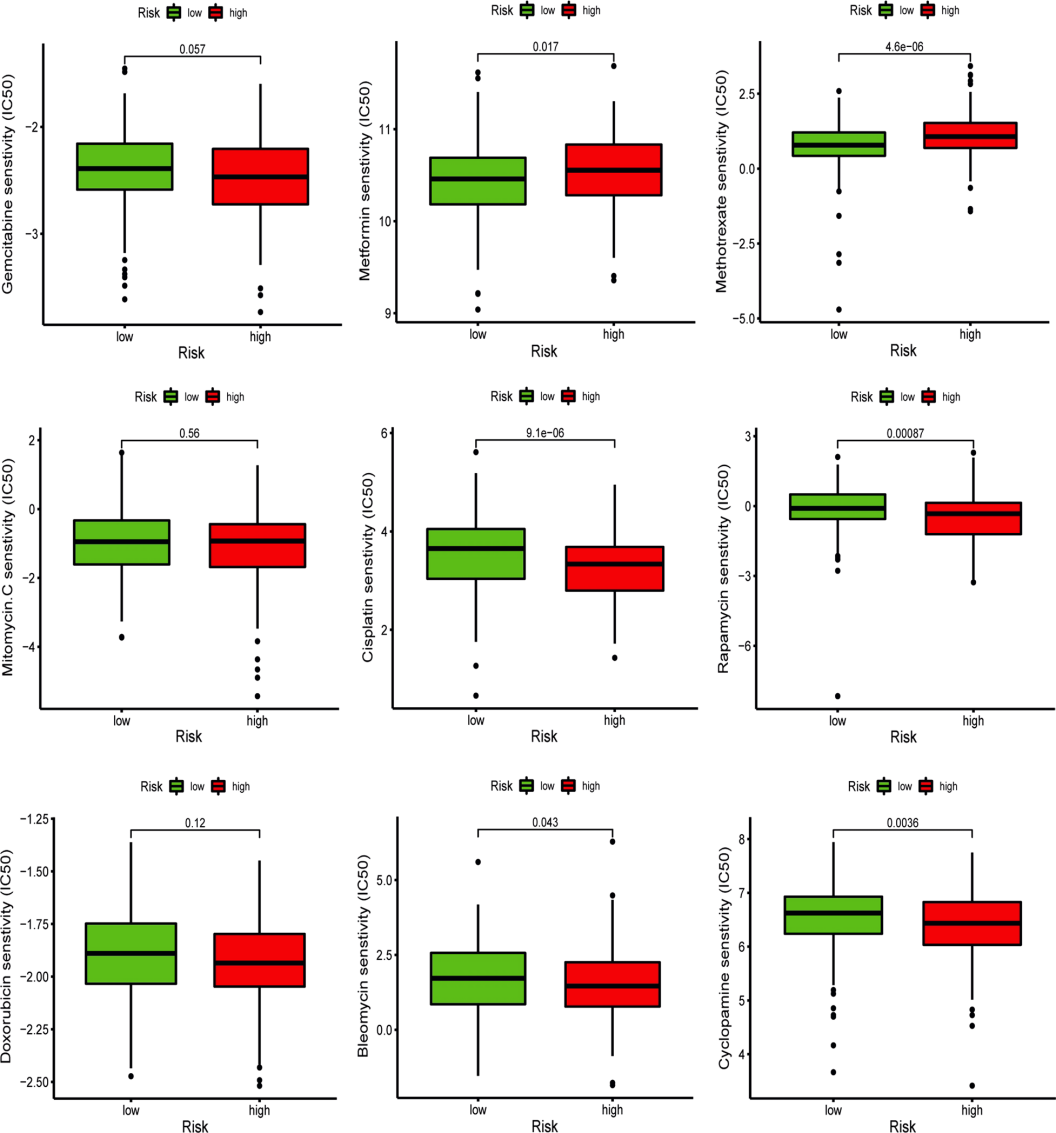
Chemotherapy sensitivity prediction between different groups

In the current research, we used GDSC database to predict the response to common chemotherapy drugs by estimating the differences of IC50 among different groups. We found that patients with high risk score were more sensitive to several chemotherapy drugs, including cisplatin, rapamycin, cyclopamine, and bleomycin, while patients with low risk score were more sensitive to metformin and methotrexate (Fig. 16). In addition, there was no obvious difference in **s**ensitivity of gemcitabine, mitomycin C, and doxorubicin among two groups.

## Discussion

Bladder cancer (BLCA) is a highly heterogeneous malignancy that develops via a complex multistep biological process. Accumulating evidence has demonstrated the crucial role of the UPR in the progression and outcome of various types of cancer, including BLCA [12, 18, 19]. The UPR was mainly mediated by three ER transmembrane protein sensors: inositol requiring kinase 1 (IRE1), protein kinase RNA-like endoplasmic reticulum kinase (PERK), and activating transcription factor 6 (ATF6) [20]. Silencing OTUB1 can markedly suppress the proliferation and migration of BLCA cells through activating ATF6 signaling [18]. Most of studies focused on the effect of UPR in cancer progression and metastasis, and few studies have investigated the prognostic value of UPR-related genes in cancers, especially in BLCA. Recently, mounting evidence has confirmed that multiple genes model is being used to predict outcomes and therapeutic effect seems to have high credibility [21–24].

Hence, in the current study, we conducted comprehensive bioinformatics approach to investigate the expression patterns of 251 UPR-related genes in BLCA and their correlation with OS. We identified a prognostic signature consisting of 7 UPRGs correlated with progression and outcomes of patients with BLCA and verified the novel prognostic signature in an external dataset GSE13507. Univariate and multivariate Cox regression analysis suggested that the signature based on 7 UPRGs was independent prognostic factor for survival in BLCA patients. Multivariate ROC analysis showed that risk scores were superior to traditional prognostic factors in predicting outcomes of patients with BLCA. In addition, UPR-based signature was closely associated with inferior clinical characteristics. Subgroups analyses stratified by clinical characteristics also indicated that patients with low risk scores had longer survival than those with high risk scores. A nomogram incorporating clinical characteristics and risk score was established to predict the outcomes of BLCA patients.

Among the seven UPRGs in the prognostic signature that we established, calreticulin (CALR), with a high ability for Ca^2+^ binding, has been implicated in the homeostasis of Ca^2+^ and protein folding as well as cell adhesion [25]. Several studies have also shown that CALR was associated with immune responses and apoptosis [26, 27]. Mounting evidence suggested that CALR expression was correlated with carcinogenesis, progression, outcome, and drug resistance as well as epithelial–mesenchymal transition [28]. Alpha-crystallin B (CRYAB), a member of the small heat-shock protein family with the functions of suppressing protein aggregation and promoting protein refolding, has been reported to inhibit the tumorigenic phenotypes of BLCA cells through suppression of the PI3K/AKT and ERK signaling pathway [29]. DnaJ homolog subfamily B member 4 (DNAJB4), also called HLJ1, has been proven to serve as a tumor suppressor role in many types of cancer including lung cancer, melanoma, and breast cancer [30–32]. Genetic deletion of KDELR3 was found to inhibit the lung metastasis of melanoma cells [33]. CREB3L3, an ER stress-associated transcription factor, was shown to be related to the proliferation of HBV-associated HCC cells by regulating the PI3K/Akt and AMPK signaling pathways [34]. Studies have confirmed the critical function of HSPB6 in the endothelial proliferation and migration of various cancers [35, 36]. Overexpression of HSPB6 suppressed BLCA T24 cell migration to a certain level but had no effect on T24 cell proliferation [37]. F-box protein 6 (FBXO6), a vital component of the ubiquitin protein ligase complex, has been reported to be involved in the invasion and sensitivity of cisplatin [38]. Wang et al. reported that FBXO6 might be a CD8^+^ T cell infiltration-promoting factor and improve anti-PD1 drug resistance in BLCA patients [39].

Previous studies have confirmed that UPR was highly correlated with immune cell infiltration. In addition, accumulating evidence has also confirmed that immune cell infiltration was closely associated with the development, progression, and prognosis as well as the treatment of BLCA. Therefore, the stromal score, ESTIMATE score, and immune score of BLCA samples were estimated by making use of the ESTIMATE algorithm. We found that the risk score was positively correlated with immune score, stromal score, and ESTIMATE score. Subsequently, we further used the CIBERSORT algorithm and ssGSEA to explore the relationship between the prognostic signature and immune cell infiltration. The results indicated that the signature based on 7 UPRGs was closely associated with immune cell infiltration. The low-risk group had a higher proportion of CD8^+^ T cells than high-risk groups, which was in line with previous research showing that the infiltration level of CD8^+^ T cells was positively related to favorable prognosis [39, 40]. In contrast, M2 macrophages and neutrophils had higher proportions in the high-risk group than low-risk group, which was also in accordance with previous studies that M2 macrophages and neutrophils were related to unfavorable outcomes [41–43]. In addition, cytokines (IL-10, TGF-β1, TGF-β2, TGF-β3) involved in immunosuppressive process and immune checkpoints (PD-L1, CTLA-4, and HAVCR2) were elevated in high-risk group, which indicated the immunosuppression microenvironment in high-risk group. These results exhibited the potential of UPR signature in predicting immune microenvironment of BLCA, which might improve the immunotherapeutic effect of tumor.

We further investigated the potential mechanisms among different groups and found that immune and carcinogenic pathways were enriched in high-risk group, which might explain the unfavorable prognosis and immune-suppressive status of patients in high-risk group, while the metabolism-associated pathways were highly enriched in low-risk groups. In addition, patients with high risk score might benefit from several chemotherapy drugs, including cisplatin, rapamycin, cyclopamine, and bleomycin, while patients with low risk score might benefit from the treatments of metformin and methotrexate.

In brief, UPR plays a critical role in the occurrence and progression of tumors. Our study demonstrated the value of a set of UPRGs as a prognostic biomarker in BLCA. Nevertheless, as a retrospective study performed through bioinformatics analyses, this study inevitably has several disadvantages. In addition, the biological mechanisms of these prognostic UPRGs remain to be further confirmed by relevant experiments and clinical evidence.

## Conclusion

In summary, we established and validated a risk model based on seven UPRGs for predicting the prognosis, immune microenvironment, and chemotherapy response of patients with BLCA, which might have reliable potential for clinical application in BLCA.

## Data Availability

The raw data of this study are derived from the TCGA database (https://portal.gdc.cancer.gov/) and GEO data portal (https://www.ncbi.nlm.nih.gov/geo/), which are publicly available databases.

## References

[1] Kamat AM, Hahn NM, Efstathiou JA, Lerner SP, Malmström P-U, Choi W (2016). Bladder cancer. The Lancet.

[2] Patel VG, Oh WK, Galsky MD (2020). Treatment of muscle-invasive and advanced bladder cancer in 2020. CA Cancer J Clin.

[3] Sidrauski C, Chapman R, Walter P (1998). The unfolded protein response: an intracellular signalling pathway with many surprising features. Trends Cell Biol.

[4] Kaufman RJ (1999). Stress signaling from the lumen of the endoplasmic reticulum: coordination of gene transcriptional and translational controls. Genes Dev.

[5] Li X, Zhang K, Li Z (2011). Unfolded protein response in cancer: the Physician’s perspective. J Hematol Oncol.

[6] Cao SS, Kaufman RJ (2012). Unfolded protein response. Curr Biol.

[7] Wang G, Yang Z-Q, Zhang K (2010). Endoplasmic reticulum stress response in cancer: molecular mechanism and therapeutic potential. Am J Transl Res.

[8] Ron D (2002). Translational control in the endoplasmic reticulum stress response. J Clin Invest.

[9] Scheuner D, Song B, McEwen E, Liu C, Laybutt R, Gillespie P (2001). Translational Control Is Required for the Unfolded Protein Response and In Vivo Glucose Homeostasis. Mol Cell juin.

[10] Mori K (2000). Tripartite management of unfolded proteins in the endoplasmic reticulum. Cell mai.

[11] Bobrovnikova-Marjon E, Grigoriadou C, Pytel D, Zhang F, Ye J, Koumenis C (2010). PERK promotes cancer cell proliferation and tumor growth by limiting oxidative DNA damage. Oncogene.

[12] Li C, Huang Y, Fan Q, Quan H, Dong Y, Nie M (2021). p97/VCP is highly expressed in the stem-like cells of breast cancer and controls cancer stemness partly through the unfolded protein response. Cell Death Dis avr.

[13] Siegelin MD, Dohi T, Raskett CM, Orlowski GM, Powers CM, Gilbert CA (2011). Exploiting the mitochondrial unfolded protein response for cancer therapy in mice and human cells. J Clin Invest.

[14] Tameire F, Verginadis II, Koumenis C (2015). Cell intrinsic and extrinsic activators of the unfolded protein response in cancer: mechanisms and targets for therapy. Semin Cancer Biol.

[15] Rufo N, Garg AD, Agostinis P (2017). The unfolded protein response in immunogenic cell death and cancer immunotherapy. Trends Cancer sept.

[16] Martelli AM, Paganelli F, Chiarini F, Evangelisti C, McCubrey JA (2020). The unfolded protein response: a novel therapeutic target in acute leukemias. Cancers.

[17] Kim W-J, Kim E-J, Kim S-K, Kim Y-J, Ha Y-S, Jeong P (2010). Predictive value of progression-related gene classifier in primary non-muscle invasive bladder cancer. Mol Cancer.

[18] Zhang H-H, Li C, Ren J-W, Liu L, Du X-H, Gao J (2021). OTUB1 facilitates bladder cancer progression by stabilizing ATF6 in response to endoplasmic reticulum stress. Cancer Sci.

[19] Hua Y-Q, Zhang K, Sheng J, Ning Z-Y, Li Y, Shi W (2021). NUCB1 suppresses growth and shows additive effects with gemcitabine in pancreatic ductal adenocarcinoma via the unfolded protein response. Front Cell Dev Biol.

[20] Madden E, Logue SE, Healy SJ, Manie S, Samali A (2019). The role of the unfolded protein response in cancer progression: from oncogenesis to chemoresistance. Biol Cell.

[21] Qi W, Zhang Q (2021). Development and clinical validation of a 3-miRNA signature to predict prognosis of gastric cancer. PeerJ.

[22] Wu Y, Liu Y, He A, Guan B, He S, Zhang C (2020). Identification of the Six-RNA-binding protein signature for prognosis prediction in bladder cancer. Front Genet.

[23] Zhang L, Chen S, Wang B, Su Y, Li S, Liu G (2019). An eight-long noncoding RNA expression signature for colorectal cancer patients’ prognosis. J Cell Biochem.

[24] Gu Y, Chen G, Lin P, Cheng J, Huang Z, Luo J (2020). Development and validation of an immune prognostic classifier for clear cell renal cell carcinoma. Cancer Biomark.

[25] Lu Y-C, Weng W-C, Lee H (2015). Functional roles of calreticulin in cancer biology. BioMed Res Int.

[26] Fujiwara Y, Tsunedomi R, Yoshimura K, Matsukuma S, Fujiwara N, Nishiyama M (2021). Pancreatic cancer stem-like cells with high calreticulin expression associated with immune surveillance. Pancreas.

[27] Fucikova J, Spisek R, Kroemer G, Galluzzi L (2021). Calreticulin and cancer. Cell Res.

[28] Kasikova L, Hensler M, Truxova I, Skapa P, Laco J, Belicova L (2019). Calreticulin exposure correlates with robust adaptive antitumor immunity and favorable prognosis in ovarian carcinoma patients. J Immunother Cancer.

[29] Ruan H, Li Y, Wang X, Sun B, Fang W, Jiang S (2020). CRYAB inhibits migration and invasion of bladder cancer cells through the PI3K/AKT and ERK pathways. Jpn J Clin Oncol.

[30] Miao W, Li L, Wang Y (2018). A targeted proteomic approach for heat shock proteins reveals DNAJB4 as a suppressor for melanoma metastasis. Anal Chem.

[31] Mo L, Liu J, Yang Z, Gong X, Meng F, Zou R (2020). DNAJB4 identified as a potential breast cancer marker: evidence from bioinformatics analysis and basic experiments. Gland Surg.

[32] Chen C-H, Chang W-H, Su K-Y, Ku W-H, Chang G-C, Hong Q-S (2016). HLJ1 is an endogenous Src inhibitor suppressing cancer progression through dual mechanisms. Oncogene.

[33] Marie KL, Sassano A, Yang HH, Michalowski AM, Michael HT, Guo T (2020). Melanoblast transcriptome analysis reveals pathways promoting melanoma metastasis. Nat Commun.

[34] Vecchi C, Montosi G, Garuti C, Corradini E, Sabelli M, Canali S (2014). Gluconeogenic signals regulate iron homeostasis via hepcidin in mice. Gastroenterology.

[35] Zhang X, Wang X, Zhu H, Kranias EG, Tang Y, Peng T (2012). Hsp20 Functions as a novel cardiokine in promoting angiogenesis via activation of VEGFR2. Qin G, rédacteur. PLoS ONE.

[36] Chen S, Huang H, Yao J, Pan L, Ma H (2014). Heat shock protein B6 potently increases non-small cell lung cancer growth. Mol Med Rep.

[37] Chen Y, Xu T, Xie F, Wang L, Liang Z, Li D (2021). Evaluating the biological functions of the prognostic genes identified by the Pathology Atlas in bladder cancer. Oncol Rep.

[38] Hong X, Huang H, Qiu X, Ding Z, Feng X, Zhu Y (2018). Targeting posttranslational modifications of RIOK1 inhibits the progression of colorectal and gastric cancers. Elife.

[39] Wang Y, Yan K, Lin J, Liu Y, Wang J, Li X (2020). CD8^+^ T cell co-expressed genes correlate with clinical phenotype and microenvironments of urothelial cancer. Front Oncol.

[40] Lin C-T, Tung C-L, Tsai Y-S, Shen C-H, Jou Y-C, Yu M-T (2012). Prognostic relevance of preoperative circulating CD8-positive lymphocytes in the urinary bladder recurrence of urothelial carcinoma. Urol Oncol Semin Orig Investig.

[41] Sharifi L, Nowroozi MR, Amini E, Arami MK, Ayati M, Mohsenzadegan M (2019). A review on the role of M2 macrophages in bladder cancer; pathophysiology and targeting. Int Immunopharmacol.

[42] Lv Y, Jin P, Chen Z, Zhang P (2020). Characterization of hazard infiltrating immune cells and relative risk genes in bladder urothelial carcinoma. Am J Transl Res.

[43] Xue Y, Tong L, Liu F, Liu A, Zeng S, Xiong Q (2019). Tumor-infiltrating M2 macrophages driven by specific genomic alterations are associated with prognosis in bladder cancer. Oncol Rep.

